# Supplementary material for: Single-cell analysis of skeletal muscle macrophages reveals age-associated functional subpopulations
Source: eLife. 2022 Oct 19;11:e77974. doi: 10.7554/eLife.77974 (PMC9629833; doi:10.7554/eLife.77974)
Supplement: Supplementary file 5. [file elife-77974-supp5.docx]

| Serial No. | Antibody | Cat No. | Clone | Company | Dilution |
| --- | --- | --- | --- | --- | --- |
| 1 | BUV395 Anti-Mouse CD45 | 564279 | 30-F11 | BDBiosciences | 1:100 |
| 2 | PE anti-mouse/human CD11b Antibody | 101208 | M1/70 | Biolegend | 1:100 |
| 3 | PE-Cyanine7 anti-mouse/ human CD11b Antibody | 101216 | M1/70 | Biolegend | 1:100 |
| 4 | PE/Cyanine7 anti-mouse F4/80 Antibody | 123114 | BM8 | Biolegend | 1:40 |
| 5 | BUV737 Anti-Mouse F4/80 | 749283 | T45-2342 (RUO) | BDBiosciences | 1:40 |
| 6 | APC Anti-Mouse Lyve1 Antibody | 50-0443-82 | ALY7 | Thermofisher | 1:20 |
| 7 | Brilliant Violet 711™ anti-mouse I-A/I-E Antibody | 107643 | M5/114.15.2 | Biolegend | 1:40 |
| 8 | BD Horizon™ Fixable Viability Stain 780 | 565388 | - | BDBiosciences | 1:4000 |
| 9 | TruStain FcX™ (anti-mouse CD16/32) | 101320 | 93 | Biolegend | 1:1000 |
| 10 | Anti-Mouse LYVE1 antibody | ab14917 | - | abcam | 1:200 |
| 11 | MHC Class II (I-A/I-E) Monoclonal Antibody | 14-5321-82 | M5/114.15.2 | Thermofisher | 1:100 |
| 12 | Anti-Integrin αM/CD11b Antibody | sc-1186 | Integrin αM (44) | Santa Cruz | 1:50 |

Table S5: List of antibodies used for this study
