# Supplementary material for: Single-cell analysis of skeletal muscle macrophages reveals age-associated functional subpopulations
Source: eLife. 2022 Oct 19;11:e77974. doi: 10.7554/eLife.77974 (PMC9629833; doi:10.7554/eLife.77974)
Supplement: Supplementary file 6. [file elife-77974-supp6.docx]

**Table S6: R processing scripts used for data analysis**

library(Seurat)

library(cowplot)

library(calibrate)

library(SingleCellExperiment)

library(dplyr)

library(mclust)

library(umap)

library(ggplot2)

library(magrittr)

library(patchwork)

#Young1

Y1 <- Read10X(data.dir = "../Macrophage_1_Young/outs/filtered_feature_bc_matrix")

y1 <- CreateSeuratObject(counts = Y1, min.cells = 10, min.features = 200)

y1[["percent.mt"]] <- PercentageFeatureSet(y1, pattern = "^mt-")

y1 <- subset(y1, subset = nFeature_RNA > 300 & nFeature_RNA < 7000 & nCount_RNA > 500 & nCount_RNA < 60000 & percent.mt < 7.5)

y1 <- subset(y1, subset = (Itgam>0 & Adgre1>0), slot="counts")

#Young2

Y2 <- Read10X(data.dir = "../Macrophage_2_Young/outs/filtered_feature_bc_matrix")

y2 <- CreateSeuratObject(counts = Y2, min.cells = 10, min.features = 200)

y2[["percent.mt"]] <- PercentageFeatureSet(y2, pattern = "^mt-")

y2 <- subset(y2, subset = nFeature_RNA > 300 & nFeature_RNA < 7000 & nCount_RNA > 500 & nCount_RNA < 60000 & percent.mt < 7.5)

y2 <- subset(y2, subset = (Itgam>0 & Adgre1>0), slot="counts")

#Young3

Y3 <- Read10X(data.dir = "../Macrophage_3_Young/outs/filtered_feature_bc_matrix")

y3 <- CreateSeuratObject(counts = Y3, min.cells = 10, min.features = 200)

y3[["percent.mt"]] <- PercentageFeatureSet(y3, pattern = "^mt-")

y3 <- subset(y3, subset = nFeature_RNA > 300 & nFeature_RNA < 7000 & nCount_RNA > 500 & nCount_RNA < 60000 & percent.mt < 7.5)

y3 <- subset(y3, subset = (Itgam>0 & Adgre1>0), slot="counts")

#Old1

O1 <- Read10X(data.dir = "../Macrophage_1_Old/outs/filtered_feature_bc_matrix")

o1 <- CreateSeuratObject(counts = O1, min.cells = 10, min.features = 200)

o1[["percent.mt"]] <- PercentageFeatureSet(o1, pattern = "^mt-")

o1 <- subset(o1, subset = nFeature_RNA > 300 & nFeature_RNA < 7000 & nCount_RNA > 500 & nCount_RNA < 60000 & percent.mt < 7.5)

o1 <- subset(o1, subset = (Itgam>0 & Adgre1>0), slot="counts")

#Old2

O2 <- Read10X(data.dir = "../Macrophage_2_Old/outs/filtered_feature_bc_matrix")

o2 <- CreateSeuratObject(counts = O2, min.cells = 10, min.features = 200)

o2[["percent.mt"]] <- PercentageFeatureSet(o2, pattern = "^mt-")

o2 <- subset(o2, subset = nFeature_RNA > 300 & nFeature_RNA < 7000 & nCount_RNA > 500 & nCount_RNA < 60000 & percent.mt < 7.5)

o2 <- subset(o2, subset = (Itgam>0 & Adgre1>0), slot="counts")

#Old3

O3 <- Read10X(data.dir = "../Macrophage_3_Old/outs/filtered_feature_bc_matrix")

o3 <- CreateSeuratObject(counts = O3, min.cells = 10, min.features = 200)

o3[["percent.mt"]] <- PercentageFeatureSet(o3, pattern = "^mt-")

o3 <- subset(o3, subset = nFeature_RNA > 300 & nFeature_RNA < 7000 & nCount_RNA > 500 & nCount_RNA < 60000 & percent.mt < 7.5)

o3 <- subset(o3, subset = (Itgam>0 & Adgre1>0), slot="counts")

###################################

y1[["orig.ident"]] <-"Young_1"

y2[["orig.ident"]] <-"Young_2"

y3[["orig.ident"]] <-"Young_3"

o1[["orig.ident"]] <-"Old_1"

o2[["orig.ident"]] <-"Old_2"

o3[["orig.ident"]] <-"Old_3"

y1[["condition"]] <-"Young"

y2[["condition"]] <-"Young"

y3[["condition"]] <-"Young"

o1[["condition"]] <-"Old"

o2[["condition"]] <-"Old"

o3[["condition"]] <-"Old"

all.samples <- merge(y1, y = c(y2, y3, o1, o2, o3), add.cell.ids=c("Young_1","Young_2","Young_3","Old_1","Old_2","Old_3"), project=("Macrophage_YO"))

all.samples <- SplitObject(all.samples, split.by = "orig.ident")

all.samples <- lapply(X = all.samples, FUN = function(x) {

x <- NormalizeData(x)

x <- FindVariableFeatures(x, selection.method = "vst", nfeatures = 2000)

})

features <- SelectIntegrationFeatures(object.list = all.samples)

anchors <- FindIntegrationAnchors(object.list = all.samples, anchor.features = features)

all.integrated <- IntegrateData(anchorset = anchors)

saveRDS(all.integrated, file = "SMM_integrated.rds")

DefaultAssay(all.integrated) <- "integrated"

all.integrated <- ScaleData(all.integrated, verbose = FALSE)

all.integrated <- RunPCA(all.integrated, verbose = FALSE)

ElbowPlot(all.integrated, ndims = 50)

all.integrated <- FindNeighbors(all.integrated, dims = 1:30)

all.integrated <- FindClusters(all.integrated, resolution = 0.3)

all.integrated <- RunUMAP(all.integrated, dims = 1:30)

orig.ident_order <- c("Young_1","Young_2","Young_3","Old_1","Old_2","Old_3")

all.integrated$orig.ident <- factor(all.integrated$orig.ident, levels = orig.ident_order)

condition_order <- c("Young","Old")

all.integrated$condition <- factor(all.integrated$condition, levels = condition_order)

#Fig.1C

DimPlot(all.integrated, reduction = "umap", label.size = 6, label=T, pt=0.3, group.by = "seurat_clusters")

#Fig.1D

DefaultAssay(all.integrated) <- "RNA"

pmarkers_0.5_0.32p <- FindAllMarkers(all.integrated, assay="RNA", only.pos = T, min.pct = 0.5, logfc.threshold = 0.32)

top6markers <- pmarkers_0.5_0.32p %>% group_by(cluster) %>% top_n(6, avg_log2FC )

DotPlot(all.integrated, features = (top6markers$gene),

dot.scale = 8, assay="RNA", cols=c("turquoise", "red")) + theme(text = element_text(size = 8), axis.text.x = element_text(face = "bold.italic")) + FontSize(x.text = 14, y.text = 12, y.title = 0, x.title = 0) + RotatedAxis()

#Fig.2A

DefaultAssay(all.integrated) <- "RNA"

FeaturePlot(all.integrated, c("Lyve1"), pt=0.5, order = T, label=F, label.size = 4) &

scale_color_gradientn(colours = c("grey", "orange", "red"))

#Fig.7C

DimPlot(all.integrated, reduction = "umap", label.size = 7, pt=0.5, label=F, group.by = "seurat_clusters", split.by = "condition")

#Fig.7D

DefaultAssay(all.integrated) <- "RNA"

FeaturePlot(all.integrated, c("Gpnmb"), pt=0.75, order = T, label=F, ncol= 2, label.size = 4, split.by="condition") &

scale_color_gradientn(colours = c("grey", "orange", "red"))

VlnPlot(obj = all.integrated, features = "Gpnmb", pt=0, split.by="condition", cols=c("royalblue3","brown3"))

FeaturePlot(all.integrated, c("Spp1"), pt=0.75, order = T, label=F, ncol= 2, label.size = 4, split.by="condition") &

scale_color_gradientn(colours = c("grey", "orange", "red"))

FeaturePlot(all.integrated, c("Fabp5"), pt=0.75, order = T, label=F, ncol= 2, label.size = 4, split.by="condition") &

scale_color_gradientn(colours = c("grey", "orange", "red"))

FeaturePlot(all.integrated, c("S100a8"), pt=0.75, order = T, label=F, ncol= 2, label.size = 4, split.by="condition") &

scale_color_gradientn(colours = c("grey", "orange", "red"))

FeaturePlot(all.integrated, c("S100a9"), pt=0.75, order = T, label=F, ncol= 2, label.size = 4, split.by="condition") &

scale_color_gradientn(colours = c("grey", "orange", "red"))

#Fig.S1A

DefaultAssay(all.integrated) <- "RNA"

FeaturePlot(all.integrated, c("Ly6g"), pt=0.5, order = T, label=F, label.size = 4) &

scale_color_gradientn(colours = c("grey", "orange", "red"))

FeaturePlot(all.integrated, c("Siglecf"), pt=0.5, order = T, label=F, label.size = 4) &

scale_color_gradientn(colours = c("grey", "orange", "red"))

#Fig.S1B

DimPlot(all.integrated, reduction = "umap", label.size = 6, pt=0.2, group.by = "orig.ident", split.by = "orig.ident") + NoLegend()

#Fig.S1C

DefaultAssay(all.integrated) <- "RNA"

FeaturePlot(all.integrated, c("Mrc1"), pt=0.5, order = T, label=F, label.size = 4) &

scale_color_gradientn(colours = c("grey", "orange", "red"))

FeaturePlot(all.integrated, c("Cd80"), pt=0.5, order = T, label=F, label.size = 4) &

scale_color_gradientn(colours = c("grey", "orange", "red"))

FeaturePlot(all.integrated, c("Cd86"), pt=0.5, order = T, label=F, label.size = 4) &

scale_color_gradientn(colours = c("grey", "orange", "red"))

#Fig.S2A

DefaultAssay(all.integrated) <- "RNA"

FeaturePlot(all.integrated, c("Lyve1"), pt=0.5, order = T, label=F, label.size = 4) &

scale_color_gradientn(colours = c("grey", "orange", "red"))

FeaturePlot(all.integrated, c("Folr2"), pt=0.5, order = T, label=F, label.size = 4) &

scale_color_gradientn(colours = c("grey", "orange", "red"))

FeaturePlot(all.integrated, c("Cd163"), pt=0.5, order = T, label=F, label.size = 4) &

scale_color_gradientn(colours = c("grey", "orange", "red"))

DimPlot(all.integrated, reduction = "umap", label.size = 6, label=T, pt=0.3, group.by = "seurat_clusters")

#Fig.S4A

DefaultAssay(all.integrated) <- "RNA"

FeaturePlot(all.integrated, c("H2-Eb1"), pt=0.5, order = T, label=F, label.size = 4) &

scale_color_gradientn(colours = c("grey", "orange", "red"))

FeaturePlot(all.integrated, c("H2-Ab1"), pt=0.5, order = T, label=F, label.size = 4) &

scale_color_gradientn(colours = c("grey", "orange", "red"))

###Fig.S10

DefaultAssay(all.integrated) <- "RNA"

FeaturePlot(all.integrated, c("Gpnmb"), pt=0.75, order = T, label=F, ncol= 1, label.size = 4, split.by="orig.ident") &

scale_color_gradientn(colours = c("grey", "orange", "red"))

FeaturePlot(all.integrated, c("Spp1"), pt=0.75, order = T, label=F, ncol= 1, label.size = 4, split.by="orig.ident") &

scale_color_gradientn(colours = c("grey", "orange", "red"))

FeaturePlot(all.integrated, c("Fabp5"), pt=0.75, order = T, label=F, ncol= 1, label.size = 4, split.by="orig.ident") &

scale_color_gradientn(colours = c("grey", "orange", "red"))

FeaturePlot(all.integrated, c("S100a8"), pt=0.75, order = T, label=F, ncol= 1, label.size = 4, split.by="orig.ident") &

scale_color_gradientn(colours = c("grey", "orange", "red"))

FeaturePlot(all.integrated, c("S100a9"), pt=0.75, order = T, label=F, ncol= 1, label.size = 4, split.by="orig.ident") &

scale_color_gradientn(colours = c("grey", "orange", "red"))

#######

cn <- all.integrated@meta.data %>% as.data.table

cn <- cn[, .N, by = c("seurat_clusters", "condition", "orig.ident")]

DefaultAssay(all.combined) <- "RNA"

FindAllMarkers(all.integrated, assay="RNA", only.pos = F, min.pct = 0.25, logfc.threshold = 0.32)

FindAllMarkers(object = all.integrated, assay="RNA", only.pos = F, min.pct = 0.5, logfc.threshold = 0.32)

FindAllMarkers(object = all.integrated, assay="RNA", only.pos = F, min.pct = 0, logfc.threshold = 0)

all.integrated$condition <- paste(Idents(object = all.integrated),sep = "_", all.integrated$condition)

Idents(object = all.integrated) <- "condition"

levels(all.integrated)

FindMarkers(all.integrated, assay="RNA", ident.1 = c("0_Old","1_Old","2_Old","3_Old","4_Old","5_Old","6_Old","7_Old","8_Old","9_Old","10_Old"),

ident.2 = c("0_Young","1_Young","2_Young","3_Young","4_Young","5_Young","6_Young","7_Young","8_Young","9_Young","10_Young"),

verbose = T, logfc.threshold = 0, min.pct = 0)

FindMarkers(all.integrated, assay="RNA", ident.1 = c("0_Old") , ident.2 = c("0_Young"), verbose = T, logfc.threshold = 0, min.pct = 0)

FindMarkers(all.integrated, assay="RNA", ident.1 = c("1_Old") , ident.2 = c("1_Young"), verbose = T, logfc.threshold = 0, min.pct = 0)

FindMarkers(all.integrated, assay="RNA", ident.1 = c("2_Old") , ident.2 = c("2_Young"), verbose = T, logfc.threshold = 0, min.pct = 0)

FindMarkers(all.integrated, assay="RNA", ident.1 = c("3_Old") , ident.2 = c("3_Young"), verbose = T, logfc.threshold = 0, min.pct = 0)

FindMarkers(all.integrated, assay="RNA", ident.1 = c("4_Old") , ident.2 = c("4_Young"), verbose = T, logfc.threshold = 0, min.pct = 0)

FindMarkers(all.integrated, assay="RNA", ident.1 = c("5_Old") , ident.2 = c("5_Young"), verbose = T, logfc.threshold = 0, min.pct = 0)

FindMarkers(all.integrated, assay="RNA", ident.1 = c("6_Old") , ident.2 = c("6_Young"), verbose = T, logfc.threshold = 0, min.pct = 0)

FindMarkers(all.integrated, assay="RNA", ident.1 = c("7_Old") , ident.2 = c("7_Young"), verbose = T, logfc.threshold = 0, min.pct = 0)

FindMarkers(all.integrated, assay="RNA", ident.1 = c("8_Old") , ident.2 = c("8_Young"), verbose = T, logfc.threshold = 0, min.pct = 0)

FindMarkers(all.integrated, assay="RNA", ident.1 = c("9_Old") , ident.2 = c("9_Young"), verbose = T, logfc.threshold = 0, min.pct = 0)

FindMarkers(all.integrated, assay="RNA", ident.1 = c("10_Old") , ident.2 = c("10_Young"), verbose = T, logfc.threshold = 0, min.pct = 0)

## Supervised

all.integrated <- readRDS(file = "SMM_integrated.rds")

DefaultAssay(all.integrated) <- "integrated"

all.integrated <- ScaleData(all.integrated, verbose = FALSE)

all.integrated <- RunPCA(all.integrated, verbose = FALSE)

ElbowPlot(all.integrated, ndims = 50)

all.integrated <- RunUMAP(all.integrated, dims = 1:30)

DimPlot(all.integrated, label=F)

DefaultAssay(all.integrated) <- "RNA"

LnHl <- subset(all.integrated, subset = (Lyve1 <= 0 & `H2-Ab1` < 2),slot="data")

all.integrated <- SetIdent(all.integrated, cells = Cells(LnHl), value = "LnHl")

LnHh <- subset(all.integrated, subset = (Lyve1 <= 0 & `H2-Ab1` >= 2), slot="data")

all.integrated <- SetIdent(all.integrated, cells = Cells(LnHh), value = "LnHh")

LpHh <- subset(all.integrated, subset = (Lyve1 > 0 & `H2-Ab1` >= 2), slot="data")

all.integrated <- SetIdent(all.integrated, cells = Cells(LpHh), value = "LpHh")

LpHl <- subset(all.integrated, subset = (Lyve1 > 0 & `H2-Ab1`< 2), slot="data")

all.integrated <- SetIdent(all.integrated, cells = Cells(LpHl), value = "LpHl")

all.integrated[["idents"]] <- Idents(object = all.integrated)

levels(all.integrated) <- c('LpHl', 'LpHh', 'LnHh', 'LnHl')

levels(x = all.integrated)

colnames(all.integrated[[]])

DimPlot(all.integrated)

orig.ident_order <- c("Young_1","Young_2","Young_3","Old_1","Old_2","Old_3")

all.integrated$orig.ident <- factor(all.integrated$orig.ident, levels = orig.ident_order)

condition_order <- c("Young","Old")

all.integrated$condition <- factor(all.integrated$condition, levels = condition_order)

#Fig.3A

DimPlot(all.integrated, reduction = "umap", label.size = 6, pt=0.5, label=F, split.by = "idents")

#Fig.3A and S4A

DimPlot(all.integrated, reduction = "umap", label.size = 6, label=F, pt=0.3)

#Fig.3B

DefaultAssay(all.integrated) <- "RNA"

markers <- FindAllMarkers(object = all.integrated, assay="RNA", only.pos = T, min.pct = 0.25, logfc.threshold = 1)

all.integrated_h <- ScaleData(object = all.integrated, features = rownames(all.integrated))

DoHeatmap(object = all.integrated_h, assay="RNA",features = markers$gene, label = TRUE) + scale_fill_gradientn(colors = c("royalblue", "gray", "red")) + theme(axis.text.y = element_text(size=2, face = "bold.italic"))

#Fig.S4B

DimPlot(all.integrated, reduction = "umap", label.size = 6, pt=0.5, label=F, split.by = "orig.ident")

## LnHl subset clustering

DefaultAssay(all.integrated) <- "RNA"

LnHl <- subset(x = all.integrated, idents = c("LnHl"))

LnHl <- NormalizeData(LnHl, normalization.method = "LogNormalize",

scale.factor = 10000, verbose = F)

LnHl <- FindVariableFeatures(LnHl, selection.method = "vst", nfeatures = 2000)

LnHl <- ScaleData(LnHl, verbose = FALSE)

LnHl <- RunPCA(LnHl, verbose = FALSE)

ElbowPlot(LnHl, ndims = 50)

LnHl <- FindNeighbors(LnHl, dims = 1:30)

LnHl <- FindClusters(LnHl, resolution = 0.3)

LnHl <- RunUMAP(LnHl, dims = 1:30)

#Fig.S7A

DimPlot(LnHl, label=F, pt=2)

FindAllMarkers(LnHl, assay="RNA", only.pos = F, min.pct = 0.25, logfc.threshold = 0.32)

#######

cn <- all.integrated@meta.data %>% as.data.table

cn <- cn[, .N, by = c("idents","condition", "orig.ident")]

FindAllMarkers(all.integrated, assay="RNA", only.pos = F, min.pct = 0.25, logfc.threshold = 0.32)

FindAllMarkers(object = all.integrated, assay="RNA", only.pos = F, min.pct = 0.5, logfc.threshold = 0.32)

FindAllMarkers(object = all.integrated, assay="RNA", only.pos = F, min.pct = 0, logfc.threshold = 0)

all.integrated$condition <- paste(Idents(object = all.integrated),sep = "_", all.integrated$condition)

Idents(object = all.integrated) <- "condition"

levels(all.integrated)

FindMarkers(all.integrated, assay="RNA", ident.1 = c("LpHh_Old") , ident.2 = c("LpHh_Young"), verbose = T, logfc.threshold = 0, min.pct = 0)

FindMarkers(all.integrated, assay="RNA", ident.1 = c("LnHh_Old") , ident.2 = c("LnHh_Young"), verbose = T, logfc.threshold = 0, min.pct = 0)

FindMarkers(all.integrated, assay="RNA", ident.1 = c("LnHl_Old") , ident.2 = c("LnHl_Young"), verbose = T, logfc.threshold = 0, min.pct = 0)

FindMarkers(all.integrated, assay="RNA", ident.1 = c("LpHl_Old") , ident.2 = c("LpHl_Young"), verbose = T, logfc.threshold = 0, min.pct = 0)
