# Supplementary material for: Single-cell analysis of skeletal muscle macrophages reveals age-associated functional subpopulations
Source: eLife. 2022 Oct 19;11:e77974. doi: 10.7554/eLife.77974 (PMC9629833; doi:10.7554/eLife.77974)
Supplement: Transparent reporting form [file elife-77974-transrepform1.docx]

***eLife’s* transparent reporting form**

We encourage authors to provide detailed information *within their submission* to facilitate the interpretation and replication of experiments. Authors can upload supporting documentation to indicate the use of appropriate reporting guidelines for health-related research (see [EQUATOR Network](http://www.equator-network.org/%20)), life science research (see the [BioSharing Information Resource](https://biosharing.org/)), or the [ARRIVE guidelines](http://www.plosbiology.org/article/info:doi/10.1371/journal.pbio.1000412) for reporting work involving animal research. Where applicable, authors should refer to any relevant reporting standards documents in this form.

If you have any questions, please consult our Journal Policies and/or contact us: [editorial@elifesciences.org](mailto:editorial@elifesciences.org).

**Sample-size estimation**

- You should state whether an appropriate sample size was computed when the study was being designed
- You should state the statistical method of sample size computation and any required assumptions
- If no explicit power analysis was used, you should describe how you decided what sample (replicate) size (number) to use

Please outline where this information can be found within the submission (e.g., sections or figure legends), or explain why this information doesn’t apply to your submission:

We provided this information in the Materials and Methods section and in the figure legends. We used 2-4 biological replicates for each experiment (see below). We believe these biological replicates are sufficient to draw meaningful conclusions. We carried out statistical analysis by using Student’s T-test. More detailed information is below:

1.	For single-cell RNA-sequencing, we analyzed macrophages from 3 young and 3 old mice as biological triplicates (Fig. 1).

2.	For validation by qRT-PCR assays, we pooled 6 young mice into 2 groups (3x2) as biological duplicates (Fig. 2). Pooling was done in order to have sufficient number of cells for RNA isolation.

3.	For validation by flow cytometric analysis, we used additional 4 young and 4 old mice as biological quadruplicates (Fig. 3).

4.	For phagocytosis assays, we pooled 9 young mice into 3 groups (3x3) as biological triplicates (Fig. 4). Pooling was done in order to have sufficient number of cells for phagocytosis assays.

**Replicates**

- You should report how often each experiment was performed
- You should include a definition of biological versus technical replication
- The data obtained should be provided and sufficient information should be provided to indicate the number of independent biological and/or technical replicates
- If you encountered any outliers, you should describe how these were handled
- Criteria for exclusion/inclusion of data should be clearly stated
- High-throughput sequence data should be uploaded before submission, with a private link for reviewers provided (these are available from both GEO and ArrayExpress)

Please outline where this information can be found within the submission (e.g., sections or figure legends), or explain why this information doesn’t apply to your submission:

We stated this information in the Materials and Methods section and the figure legends. Briefly, we carried out pilot studies for all experiments first. We observed consistent results in real experiments without obvious outliers. We included all data without for exclusion/inclusion in figures and supplementary tables. We have submitted our Single-cell RNA-sequencing data to GEO (#GSE195507). More detailed information is shown below:

1.	Single-cell RNA-sequencing: We established FACS protocol first, as shown in our earlier report (Krasniewski et al, Bio-101, 2021). We analyzed 3 young and 3 old mice separately as biological triplicates. We sorted macrophages from the 6 mice in 3 separate dates in 2 weeks.

2.	RT-qRT validation: We pooled macrophages from 6 young mice into 2 groups (3x2) as biological duplicates. RT-qPCR analysis was done in two separate dates.

3.	Flow cytometric validation: We used 4 young and 4 old mice as biological quadruplicates. We analyzed 2 mice per day. 8 mice took 2 weeks.

4.	Phagocytosis assays: We pooled 9 young mice into 3 groups (3x3) as biological triplicates. This experiment was done in 2 separate dates within a week.

**Statistical reporting**

- Statistical analysis methods should be described and justified
- Raw data should be presented in figures whenever informative to do so (typically when N per group is less than 10)
- For each experiment, you should identify the statistical tests used, exact values of N, definitions of center, methods of multiple test correction, and dispersion and precision measures (e.g., mean, median, SD, SEM, confidence intervals; and, for the major substantive results, a measure of effect size (e.g., Pearson's r, Cohen's d)
- Report exact p-values wherever possible alongside the summary statistics and 95% confidence intervals. These should be reported for all key questions and not only when the p-value is less than 0.05.

Please outline where this information can be found within the submission (e.g., sections or figure legends), or explain why this information doesn’t apply to your submission:

We stated statistical analysis methods in the Materials and Methods section, and included raw data and p-values in corresponding figures. More detailed information is shown below.

1.	Single-cell RNA-sequencing: R with the Seurat package, version 4.0.4.

2.	RT-qPCR validation: Student T-test. Values represent Mean ± SD. N = 2 (biological replicates).

3.	Flow cytometric validation: Student T-test. Values represent Mean ± SD. N = 4 (biological replicates).

4.	Phagocytosis assays: Student T-test. Values represent Mean ± SD. N = 3 (biological replicates).

(For large datasets, or papers with a very large number of statistical tests, you may upload a single table file with tests, Ns, etc., with reference to sections in the manuscript.)

**Group allocation**

- Indicate how samples were allocated into experimental groups (in the case of clinical studies, please specify allocation to treatment method); if randomization was used, please also state if restricted randomization was applied
- Indicate if masking was used during group allocation, data collection and/or data analysis

Please outline where this information can be found within the submission (e.g., sections or figure legends), or explain why this information doesn’t apply to your submission:

We included this information in the Materials and Methods section and the figure legends. Briefly, we grouped mice for two specific experiments to have sufficient number of macrophages for corresponding studies as follows:

1.	RT-qPCR validation: We pooled 6 same-sex, same-age young mice randomly into 2 groups (3x2) as biological duplicates (Fig. 2).

2.	Phagocytosis assays: We pooled 9 same-sex, same-age young mice into 3 groups (3x3) randomly as biological triplicates (Fig. 4).

**Additional data files (“source data”)**

- We encourage you to upload relevant additional data files, such as numerical data that are represented as a graph in a figure, or as a summary table
- Where provided, these should be in the most useful format, and they can be uploaded as “Source data” files linked to a main figure or table
- Include model definition files including the full list of parameters used
- Include code used for data analysis (e.g., R, MatLab)
- Avoid stating that data files are “available upon request”

Please indicate the figures or tables for which source data files have been provided:

We have provided additional raw data in 5 supplementary tables with parameters used (Tables S1-S5). We believe these tables provide all the necessary information and can be easily accessed and used by the readers.
